# The Renaissance of *Pathophysiology*—The Flagship Journal of the International Society for Pathophysiology

**DOI:** 10.3390/pathophysiology27010004

**Published:** 2020-12-06

**Authors:** J. Steven Alexander

**Affiliations:** Department of Molecular and Cellular Physiology, LSU Health Shreveport, Shreveport, LA 71103, USA; JAlexa@lsuhsc.edu

The recent transfer of *Pathophysiology* (ISSN 1873-149X), the flagship journal of the *International Society for Pathophysiology* (ISP) [1], from Elsevier [2] to MDPI [3] was a jubilant occasion for the ISP and the editors of *Pathophysiology*. Our editorial and publishing teams appreciate the help of Elsevier publishing in making this a very smooth transition. We would also like to thank Dr. Olga Pechanova, the current president of the ISP, for her significant efforts in securing these transfer processes.

Our journal was initially inaugurated by Prof. Toshikazu Yoshikawa of the Kyoto Prefectural University of Medicine, Department of Molecular Gastroenterology and Hepatology, in 1994 as a quarterly journal with Elsevier with the participation of Dr. D. Neil Granger, Dr. Osmo Hänninen and Dr. Osamu Matsuo as regional editors. With MDPI, we hope to continue this legacy of research, scholarship and communication. As such, *Pathophysiology* remains an international, “open access” and peer-reviewed journal that provides a progressive platform for publishing studies related to the mechanistic basis, disease characteristics and therapies for diverse diseases. The emphasis of our journal’s title *Pathophysiology* is inherently extensive as a “discipline” and anticipates that published reports will include diagnostic, prognostic and mechanistic studies on disease, including novel therapeutic approaches and therapeutics.

Our now (hopefully) post-COVID era is a critical historical breakwater. We are entering a period with many new challenges in terms of disease detection as well as population-based and personalized/precision medical treatments where the weight of *Pathophysiology* cannot be overemphasized. As we take these first steps to redefine the scope of our journal with an emphasis on *rapidly publicizing* the newest and most highly important works in this field, we will seek to maintain a high level of familiarity in terms of editorial sophistication to allow such expedited reviewing. Therefore, we have now expanded the range of the disciplines of our editorial board members working in science and medicine, and have empanelled a large and extensive board of specialists drawn from many different disciplines to participate in reviewing submitted content. Consequently, we now draw on a large pool of active physiologists, neurologists, immunologists and pathologists with many cumulative decades of experience at the bench and in peer-reviewing and publishing such work to provide rapid responses to bring the best work out very rapidly.

The ISP also represents a worldwide network of scientists, many of whom are in Europe and are interested in creating inter-institutional and cross-disciplinary collaborations. As resources for science become increasingly more precious, we hope that participation in the ISP and submission to our journal become a regular endeavor. We invite all interested basic and clinical scientists to submit their best and boldest reports to *Pathophysiology*.
